# The role of unsaturated fatty acids in modulating human butyrylcholinesterase activity: insights from kinetics and molecular docking

**DOI:** 10.1007/s00210-025-04065-3

**Published:** 2025-03-21

**Authors:** Muslum Gok, Cigdem Cicek, Suat Sari, Ebru Bodur

**Affiliations:** 1 Department of Biochemistry, Faculty of Medicine, Mugla Sitki Kocman University, 48000 Mugla, Turkey; 2 Department of Biochemistry, Faculty of Medicine, Yuksek Ihtisas University, 06520 Ankara, Turkey; 3 Department of Pharmaceutical Chemistry, Faculty of Pharmacy, Hacettepe University, 06100 Ankara, Turkey; 4 Department of Biochemistry, Faculty of Medicine, Hacettepe University, 06100 Ankara, Turkey

**Keywords:** Enzyme kinetic, Lipid metabolism, Butyrylcholinesterase, Unsaturated fatty acids, Molecular docking

## Abstract

Butyrylcholinesterase is an abundant detoxification enzyme in human serum that is mainly synthesized in the liver. It plays a crucial role in the hydrolysis of a variety of choline esters and xenobiotics, and there is emerging evidence that it is also involved in lipid metabolism. In this study, the inhibitory effects of the major unsaturated fatty acids — arachidonic acid (AA), linoleic acid (LA), oleic acid (OA), and alpha-linolenic acid (α-LA) — on human BChE are investigated using enzyme kinetics experiments and molecular modeling analyses. These fatty acids, integral components of membrane phospholipids, differ in chain length and degree of unsaturation, which influence their inhibitory effect on BChE. Our results showed that AA had the highest IC₅₀ value of 611 µM against BChE, followed by OA, α-LA, and LA. All fatty acids showed noncompetitive inhibition, in contrast to AA, which displayed uncompetitive inhibition. Inhibitory constants (Ki) showed that OA had the strongest binding affinity due to its lowest Ki value of 321.4 µM, followed by AA, α-LA, and LA. Molecular modeling supported the in vitro results. The fatty acids were predicted to bind to a newly proposed allosteric site on BChE. Our results demonstrate that the number and position of double bonds in the alkenyl chains of fatty acids significantly influence their interactions with BChE, providing new insights into how dietary lipids regulate the enzyme. This study offers a foundation for further exploration of BChE’s role in lipid metabolism and its implications for neurodegenerative and metabolic diseases.

**Graphical abstract:**

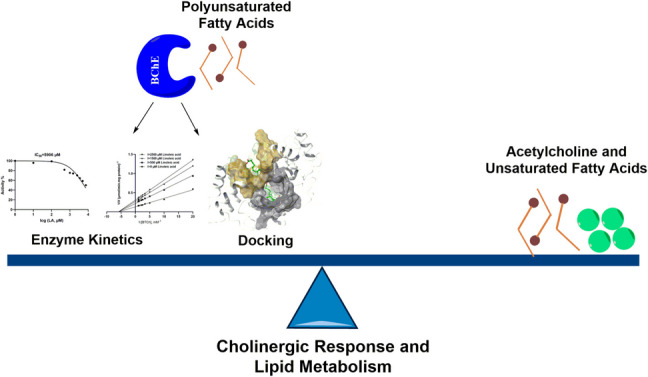

## Introduction

Butyrylcholinesterase (BChE, E.C. 3.1.1.8) is a crucial detoxification enzyme abundant in serum, capable of hydrolyzing various choline esters, ester-containing compounds, and o-nitroacetanilide (Brown et al. 1981; Montenegro et al. 2008; Masson and Lockridge 2010). Although the natural substrate of BChE has not yet been identified, its enzymatic activity is utilized for the activation of prodrugs (e.g., terbutaline) (Tunek et al. 1988) and the metabolism of xenobiotics (e.g., cocaine) (Gorelick 1997). Recent findings indicate BChE’s additional capability in lipid hydrolysis (Gok et al. 2023). Synthesized primarily in the liver, BChE constitutes approximately 0.01% of total serum proteins in humans (Ryhänen 1983). Due to mutations affecting its activity, BChE levels are often assessed prior to anesthesia administration (Lockridge and Masson 2000). Serum BChE also forms a natural complex with albumin, exhibiting a half-life of 12 days (Østergaard et al. 1988; Masson 1989). Despite sharing 65% amino acid sequence similarity with AChE, BChE differs notably in terms of selective inhibitors (Çokuğraş, 2003).

Cholinesterases, belonging to the carboxylic ester hydrolase class (E.C.3.1.1), share structural similarities with lipases and employ comparable catalytic mechanisms, suggesting potential involvement in lipid metabolism. Studies have shown that dietary fats rich in unsaturated fatty acids can influence erythrocyte membrane morphology and the activity of membrane-bound enzymes like AChE (Vajreswari and Narayanareddy 1992). Additionally, research indicates that essential fatty acids such as linoleic acid (LA) and alpha-linolenic acid (α-LA), which must be obtained through diet, may impact BChE activity, with choline analogs of LA and arachidonic acid (AA) exhibiting inhibitory effects on cholinesterase enzymes (Bulut et al. 2013; Gok et al. 2016; Akimov et al. 2020).

**Fig. 1:**
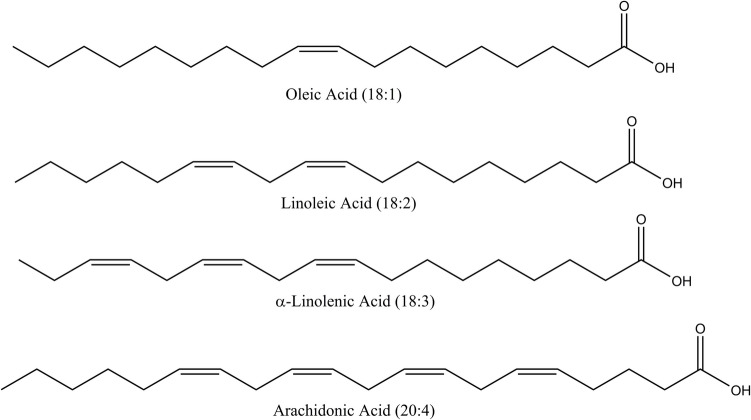
Fatty acids looked for the docking and kinetic study on human BChE

The fatty acids LA and α-LA, investigated in these studies, are integral components of membrane phospholipids. Arachidonic acid can either be synthesized from LA or liberated from membrane phospholipids via phospholipase A2 catalysis (Warude et al. 2006; Martin et al. 2016). AA is a 20-carbon unsaturated fatty acid, whereas LA and α-LA are 18-carbon unsaturated fatty acids (see Fig. 1). While LA contains 2 double bonds in its structure, α-LA possesses 3 double bonds, and AA harbors 4 double bonds. The positioning of the final double bond categorizes α-LA as omega-3 fatty acid, whereas LA and AA are classified as omega-6 fatty acids. These fatty acids serve numerous critical functions, including cell growth, inflammatory response modulation, and regulation of immune functions (Warude et al. 2006). Studies also suggest the potential benefits of omega-3 and omega-6 fatty acids in neuroinflammatory and neurodegenerative conditions such as Alzheimer’s and Parkinson’s diseases (Navarro-Mabarak et al. 2018).

Oleic acid (OA) is both obtained through dietary intake and synthesized via endogenous pathways (Schwingshackl and Hoffmann 2012). OA stands out as the predominant monounsaturated fatty acid (MUFA) in human diets, primarily formed endogenously via stearoyl-CoA desaturase 1 (SCD1) catalyzing Δ9 desaturation, chiefly from stearic acid (C18:0) (Piccinin et al. 2019). It represents a significant constituent of brain membrane phospholipids and is particularly abundant in myelin (Martínez and Mougan 1998). Remarkably, reductions in OA levels have been observed in the brains of individuals with major depressive disorders and Alzheimer’s disease (Hamazaki et al. 2012).

This study delves deeper into the inhibitory impacts of arachidonic acid, linoleic acid, oleic acid, and alpha-linolenic acid (Fig. 1) on human BChE enzyme by enzyme kinetics experiments and molecular modeling analysis. Since the enzyme kinetics suggested noncompetitive or uncompetitive inhibition by the fatty acids, we analyzed human BChE for potential ligand binding sites apart from the active site in silico and identified a possible allosteric site likely to be responsible for BChE inhibitory mechanism of the fatty acids. Molecular docking was performed to understand the molecular determinants underlying the inhibitory mechanisms of BChE by the fatty acids at molecular level. Our findings indicate variations in the influence of these fatty acids on human BChE, dependent on the length and quantity of double bonds within their alkenyl chains.

## Material and methods

### Purification of plasma BChE enzyme

The butyrylcholinesterase purification process was performed using 0 Rh (−) human blood serum from the Hacettepe Hospital blood bank, as described in a previous study (Gok et al. 2023). BChE was subjected to acid dialysis followed by DEAE-Trisacryl M ion-exchange chromatography under pH 5.5 conditions. The pooled active fractions from Trisacryl M chromatography were dialyzed to adjust neutral buffer pH 7.4 and transferred to procainamide-Sepharose 4B chromatography. The BChE enzyme was bound to the procainamide column, which was then washed with 25 mM phosphate buffer at pH 7.4. The BChE was eluted from the column with a linear gradient of 0.2–0.8 M NaCl in 25 mM potassium phosphate buffer pH 7.4, followed by dialysis. The BChE purified from the most active fractions was used for subsequent experiments. The purity of BChE was confirmed by native polyacrylamide gel electrophoresis (PAGE) as shown in a previous study (Gok et al. 2023). Silver staining (Blum et al. 1987) and the Karnovsky-Roots technique (Karnovsky and Roots 1964) were used to visualize gels that had been labeled for activity and protein, respectively.

### Determination IC₅₀ of the fatty acids

The IC₅₀ was determined using the modified 96-microtiter plate Ellman assay (George and Abernethy 1983), using a total volume of 200 µL. The fatty acids used for the kinetics were oleic acid, linoleic acid, α-linolenic acid, and arachidonic acid. The unsaturated fatty acids used for the test were dissolved in DMSO and then the working solutions were sonicated in a reaction buffer for 30 s, followed by a further 30 s. The fatty acid solutions with different final concentrations of 10–7500 µM were added to the wells. Subsequently, 50 mM MOPS buffer (pH 7.4) and 0.7 µg of purified BChE enzyme were added. The plate was incubated in the absence of light with a plate shaker at 37 °C for 10 min. Measurements were initiated by adding a final concentration of 0.25 mM DTNB (ε412, DTNB = 14.2 mM^−1^ cm^−1^) and 0.5 mM butyrylthiocholine (BTCh). The mixture of 1% DMSO without the BTCh substrate was used as the reagent blank. The absorbance readings were recorded at a wavelength of 412 nm for a period of 10 min using the Spectramax M2 microplate reader (Molecular Devices, USA). The activity without fatty acids was set as 100% control, while all activities with fatty acids were compared to this control. IC₅₀ values were determined using GraphPad Prism 8.4 software by plotting the percentage of BChE activity against the fatty acid concentration. Each test was performed at least four times and average values were reported as final results.

### Inhibition kinetics of the fatty acids

Evaluation of BChE activity was performed according to the Ellman method (George and Abernethy 1983) with a minor modification in a 96-well microtiter plate assay. The fatty acids oleic acid, linoleic acid, α-LA, and arachidonic acid used in the assay were dissolved in DMSO. The working solutions were then sonicated in a reaction buffer for 30 + 30 s. Based on previous experimental results, three concentrations of oleic acid, linoleic acid, α-LA, and arachidonic acid were chosen: 200–750 µM, 500–2500 µM, 50–250 µM, and 100–300 µM, respectively. This was to clarify the inhibitory mechanisms for the fatty acids. In the presence of these fatty acids, BChE activity was measured at eight progressively lower concentrations (1.0–0.05 mM) of the BTCh substrate. The experimental procedure involved incubation of a solution of the fatty acid with BChE enzyme (0.7 µg), 50 mM MOPS buffer pH 7.4, and 0.25 mM DTNB in the absence of light at 37 °C for 5 min. The reaction was initiated and monitored for 10 min at 412 nm in kinetic mode after addition of the substrate. The identical solution was run with buffer instead of fatty acids as a control and all assays were performed in triplicate. Enzyme activity was expressed in milliunits (mU), where 1 mU corresponds to the hydrolysis of 1 nmol of BTCh per minute at room temperature.

### Molecular docking

Ligands were modeled using LigPrep (2019-4, Schrödinger LLC, New York, NY) and MacroModel (2019-4, Schrödinger LLC, New York, NY) according to the OPLS (2019-4, Schrödinger LLC, New York, NY) forcefield parameters (Harder et al. 2016). The crystallographic structure of human BChE (PDB code: 6I0B (Chalupova et al. 2019), resolution: 2.38 Å) was downloaded from the protein data bank (www.rcsb.org) and prepared using the Protein Preparation Wizard of Maestro (2019-4, Schrödinger LLC, New York, NY) (Madhavi Sastry et al. 2013), where unwanted molecules were removed, H atoms were added, bond orders and H bonds were assigned. Ligand binding sites were detected using SiteMap (2019-4, Schrödinger LLC, New York, NY) (Halgren 2009). Molecular docking was performed using Glide (2019-4, Schrödinger LLC, New York, NY) (Friesner et al. 2006) at extra precision mode with 100 runs per ligand, docking score of the best pose for each ligand was noted. MM-GBSA calculations were performed for the top five poses of each ligand using Prime (2019-4, Schrödinger LLC, New York, NY) (Jacobson et al. 2004). Residues at 4 Å of each docked ligand were allowed flexibility; ligand-receptor complex sampling was performed using minimization according to the OPLS forcefield parameters. Free binding energy (∆G) for each ligand-receptor complex was noted. Molecular docking was validated by redocking the co-crystallized inhibitor in 6I0B and comparing its predicted pose to the original co-crystallized binding mode. The comparison was expressed statistically by calculating the root mean square deviation (RMSD) value of the predicted pose with respect to the original pose. This value was 0.97 Å and showed that the method predicted similar binding for the inhibitor with respect to its experimental binding, indicating a good predictive capacity.

### Statistical analysis

Enzyme kinetics data was analyzed and graphs were plotted using GraphPad Prism 8.42. The kinetic parameters and the enzyme-substrate relationship were fitted to the Michaelis-Menten model using nonlinear regression.

## Results

The specific enzymatic activity after purification of human BChE was 7.35 units per milligram of protein. The overall yield of the entire purification process was 33.5%, resulting in a significant enhancement in purity by approximately 300-fold (Gok et al. 2023). Both the protein and activity staining provided conclusive evidence that the purified aliquot of butyrylcholinesterase (BChE) enzyme is electrophoretically pure, further supporting the results reported in previous research (Gok et al. 2023).

**Fig. 2:**
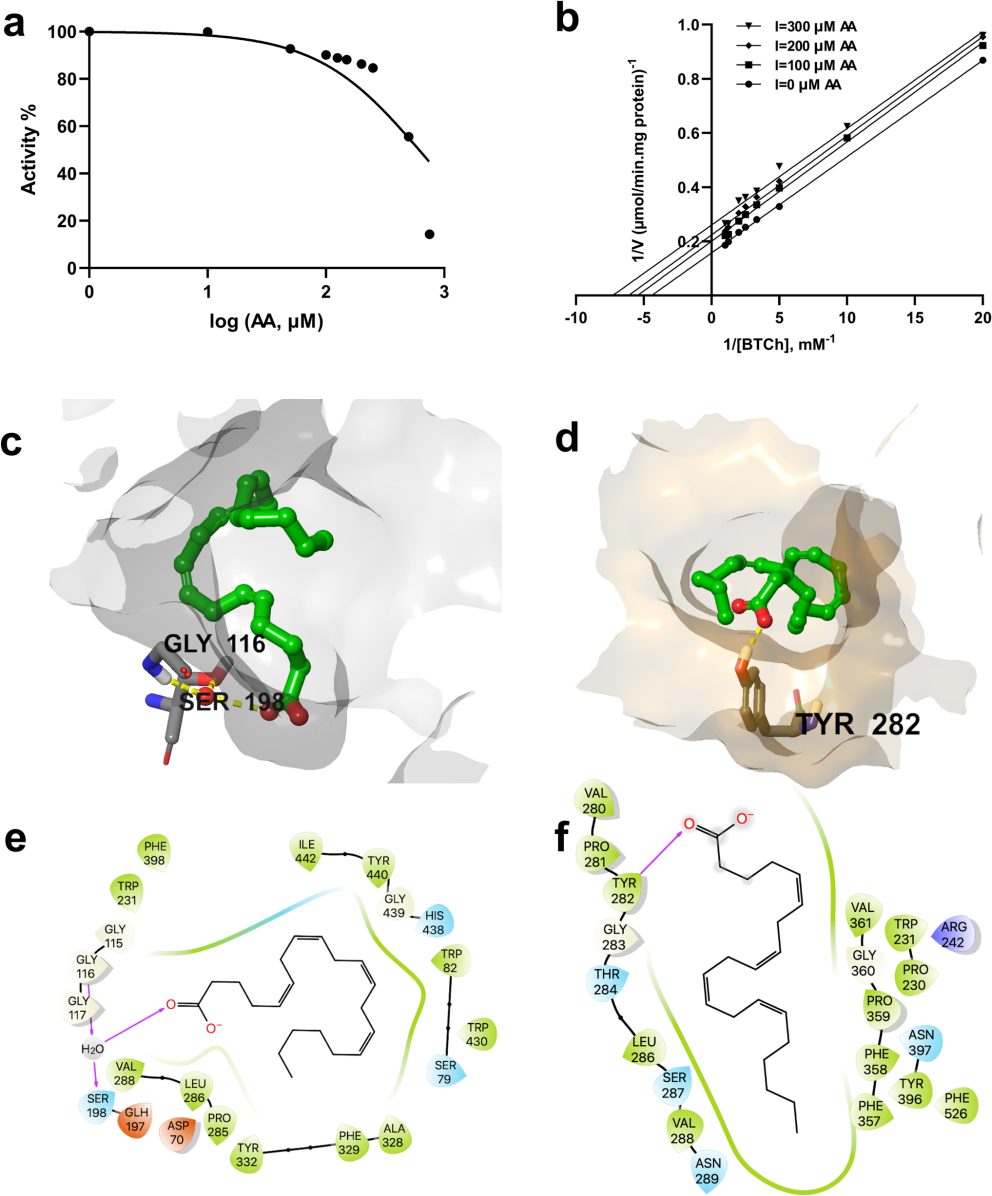
Kinetic characteristics and predicted binding interactions of arachidonic acid with BChE. The noncompetitive inhibition of BChE by AA results in an IC₅₀ of 611 µM and a Ki of 465.4 µM (**a**) and noncompetitive inhibition (**b**) of BChE by arachidonic acid. Predicted binding of arachidonic acid in the active site (**c**) and predicted allosteric site (**d**) of BChE (arachidonic acid is shown in green ball-and-stick representation, amino acid residues as gray sticks, water molecules as red spheres, and H bond interactions as yellow dashed lines). 2D interaction diagrams of arachidonic acid for its predicted binding modes with BChE (**e** and **f**, respectively)

IC₅₀ values and kinetic properties of oleic acid, linoleic acid, α-LA, and arachidonic acid were determined. Using a range of doses (10–750 µM), the IC₅₀ experiment for AA was completed. The resulting activity % vs. concentration graph revealed an IC₅₀ value of 611.3 µM (Fig. 2a). The inhibitory capacity of AA was examined at three concentrations (10–300 µM). The Lineweaver-Burk plot analysis yielded a strong fit (*R*^2^ = 0.9817) between AA concentration and enzyme inhibition (Fig. 2b). The graph demonstrates uncompetitive inhibition, where AA reduced both Vmax and Km, with a Ki of 465.4 µM and a Km of 297.3 µM. Consistent kinetic and docking data revealed a preference for AA acid to bind the allosteric site of BChE over its active site (Fig. 2c, d), exhibiting a stronger affinity for the allosteric site (∆G − 41.6) compared to the active site (∆G − 9.0) (Table 1).

**Table 1:** Docking scores (kcal/mol) and ∆G values (kcal/mol) of the fatty acids in the active and allosteric site of BChE

Compound	Active site		Allosteric site	
	Docking score	∆G	Docking score	∆G
Oleic acid	− 4.2	− 12.1	− 2.1	− 34.9
Linoleic acid	− 4.3	− 7.8	− 2.3	− 38.1
α-Linolenic acid	− 3.7	− 10.9	− 2.2	− 49.5
Arachidonic acid	− 5.9	− 9.0	− 2.8	− 41.6
BCh	− 4.1	− 36.2	-	-

**Fig. 3:**
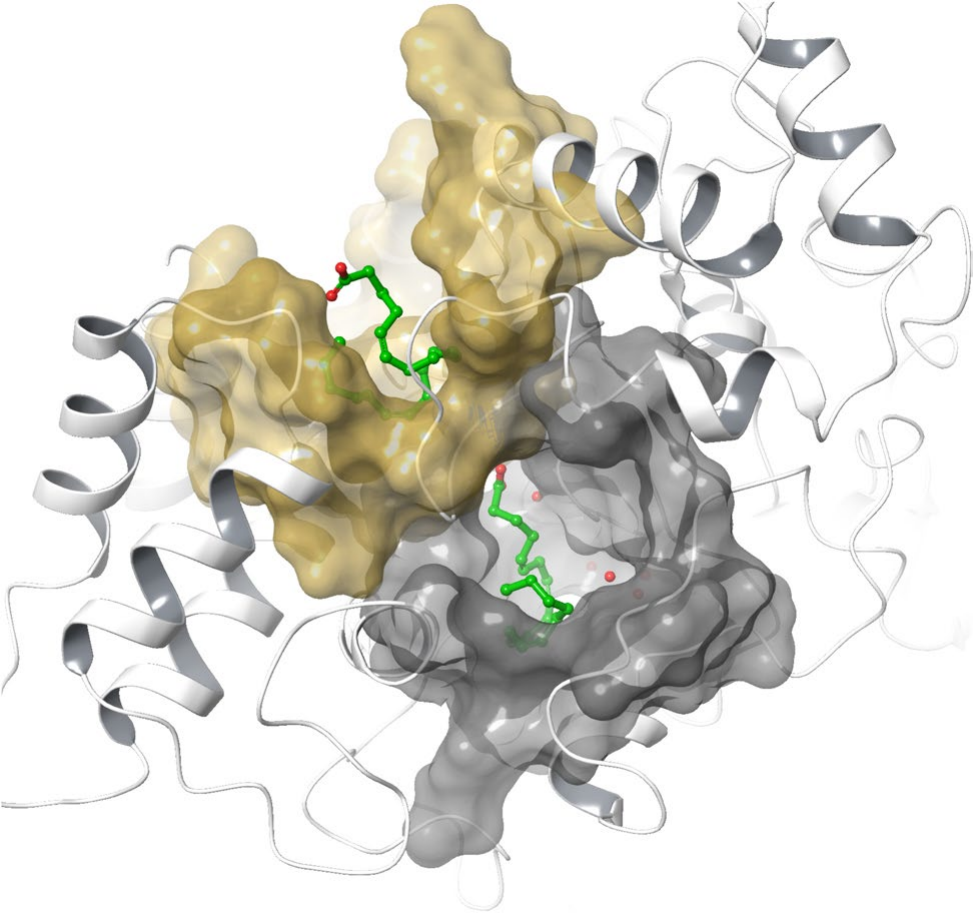
BChE (with backbone represented as white ribbons), its active site (with gray molecular surface), and the predicted allosteric ligand binding site (with a yellow molecular surface) including the predicted binding modes of arachidonic acid (green ball and stick)

According to our in silico site mapping study, BChE has another potential ligand binding site (allosteric site) located next to the active site rim, which is, more lipophilic and slightly smaller than the active site (Fig. 3). The allosteric site is comprised of the residues Phe 227, Asn 228, Ala 229, Pro 230, Trp 231, Val 233, Thr 234, Ser 235, Leu 236, Tyr 237, Glu 238, Arg 242, Val 280, Pro 281, Tyr 282, Thr 284, Leu 286, Ser 287, Val 288, Met 302, Pro 303, Asp 304, Leu 307, Glu 308, Phe 357, Phe 358, Pro 359, Gly 360, Val 361, Tyr 396, Asn 397, Cys 400, Pro 401, Glu 404, Lys 408, Trp 522, Thr523, Phe 526, and Pro 52. The BChE active site, on the other hand, consists of the residues Asn 68, Ile 69, Asp 70, Gln 71, Ser 79, Trp 82, Asn 83, Pro 84, Gly 115, Gly 116, Gly 117, Gln 119, Thr 120, Leu 125, Tyr 128, Gln 197, Ser 198, Ser 224, Trp 231, Glu 276, Ala 277, Pro 285, Leu 286, Ser 287, Val 288, Asn 289, Glu 325, Ala 328, Phe 329, Tyr 332, Phe 398, Trp 430, His 438, Gly 439, and Tyr 440. Since two sites are adjacent, they share three common residues: Leu 286, Ser 287, and Val 288.

Consistent kinetic and docking data revealed a preference for AA acid to bind the allosteric site of BChE over its active site (Fig. 2c, d), exhibiting a stronger affinity for the allosteric site (∆G =  − 41.6 kcal/mol) compared to the active site (∆G =  − 9.0 kcal/mol) (Table 1).

**Fig. 4:**
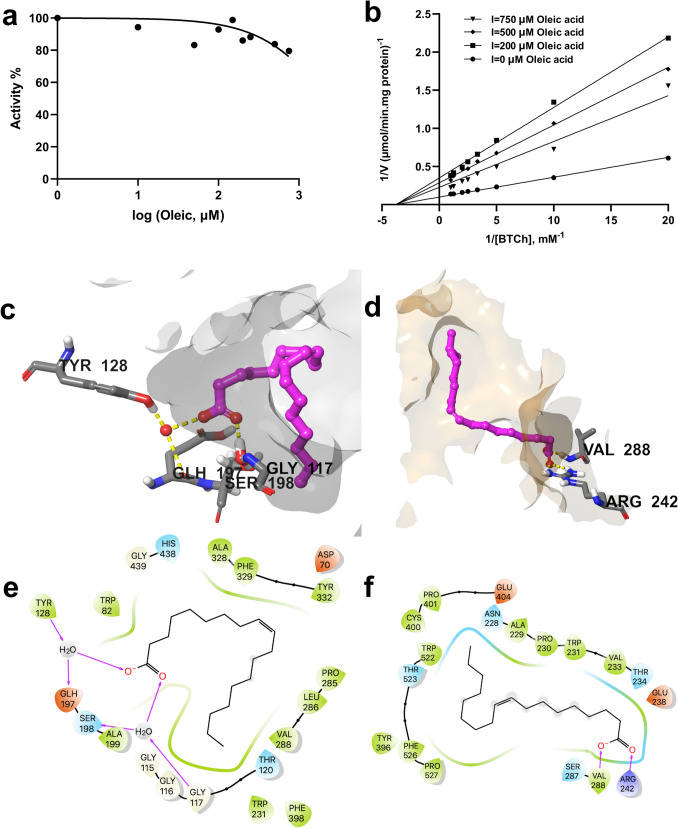
Kinetic characteristics and predicted binding interactions of oleic acid with BChE. Oleic acid inhibits BChE noncompetitively, with an IC₅₀ value of 2373 µM and a Ki of 321.4 µM (**a**, **b**). Predicted binding of oleic acid in the active site (**c**) and predicted allosteric site (**d**) of BChE (oleic acid is shown in magenta ball-and-stick representation, amino acid residues as gray sticks, water molecules as red spheres, and H bond interactions as yellow dashed lines). 2D interaction diagrams of oleic acid for its predicted binding modes with BChE (**e** and **f**, respectively)

The relationship between oleic acid concentration and %BChE activity was examined using 0.5 mM BTCh as substrate, with oleic acid concentrations ranging from 10 to 750 µM. The results are shown in Fig. 4a. The IC₅₀ value of oleic acid was calculated from the graph to be 2373 µM. To gain insight into the inhibition mechanisms of oleic acid, the activity of BChE was examined in the presence of three concentrations of oleic acid (200–750 µM), varying BTCh as substrate. The inhibition kinetics of oleic acid for BChE was illustrated in Fig. 4b. The simple linear regression of Michealis-Menten was plotted as a Lineweaver-Burk diagram and the goodness of fit was calculated as *R*^2^ = 0.957. The kinetic behavior of oleic acid showed a noncompetitive inhibitory behavior by lowering the Vmax values while Km remained unaffected. The calculated Km and Ki values for oleic acid were 264.6 µM and 321.4 µM, respectively.

Noncompetitive inhibition occurs when the molecules (oleic acid) do not compete directly with the substrate for the active site of the enzyme but bind to another site of the enzyme. Our docking results support our kinetic result showing that oleic acid binds to the allosteric site of BChE (Fig. 4c, d). Oleic acid was found to have a greater affinity for a hydrophobic pocket of the allosteric site when the ∆G values for these poses were further calculated, which were found − 34.9 and − 12.1 kcal/mol for the allosteric site and the active site of the BChE enzyme, respectively (Table 1).

**Fig. 5:**
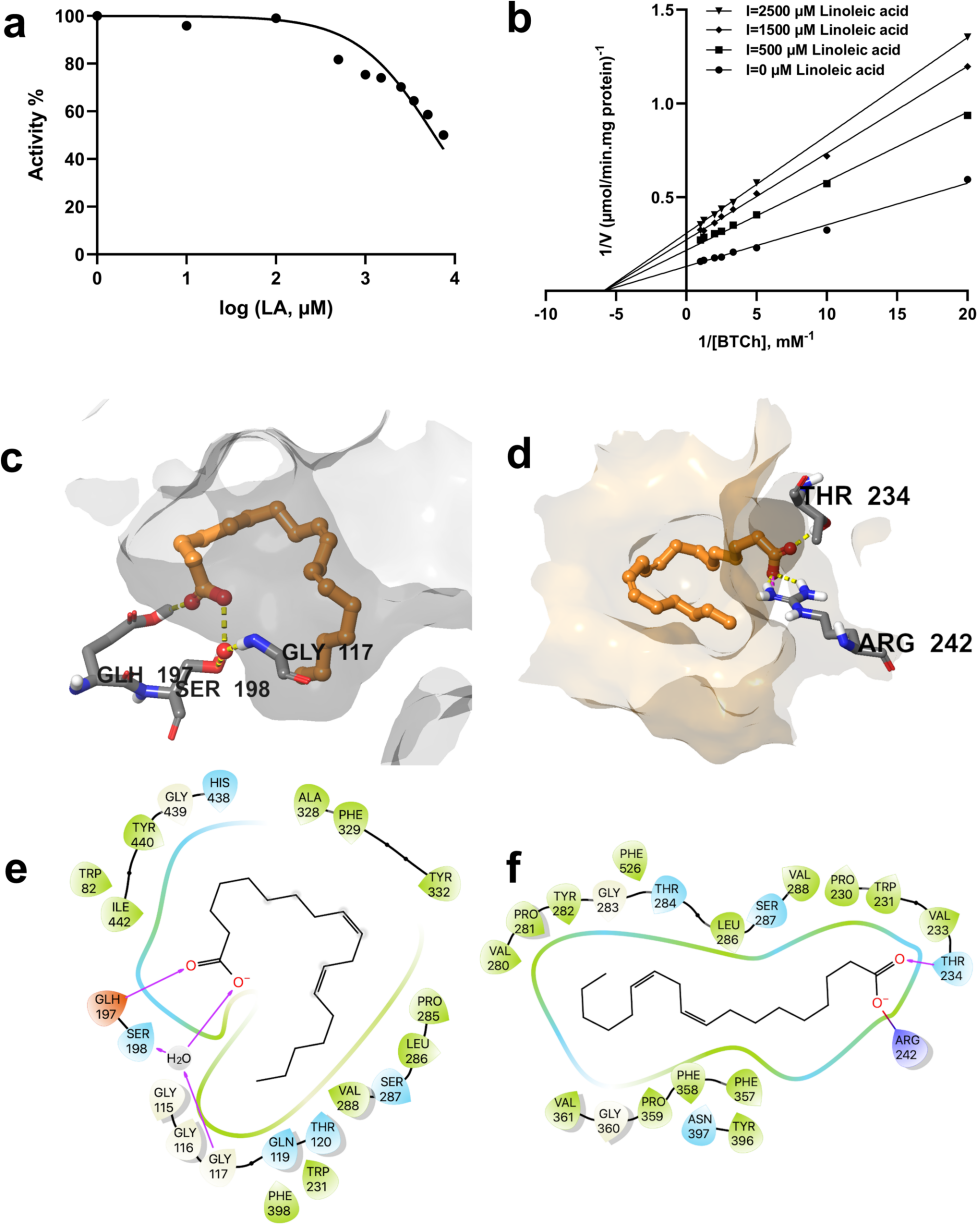
Kinetic characteristics and predicted binding interactions of linoleic acid with BChE. Noncompetitive inhibition of BChE by linoleic acid has an IC₅₀ value of 5906 µM and a Ki of 1400 µM (**a**, **b**). Predicted binding of linoleic acid in the active site (**c**) and predicted allosteric site (**d**) of BChE (linoleic acid is shown in orange ball-and-stick representation, amino acid residues as gray sticks, water molecules as red spheres, and H bond interactions as yellow dashed lines). 2D interaction diagrams of linoleic acid for its predicted binding modes with BChE (**e** and **f**, respectively)

Different concentrations of linoleic acid (10–7500 µM) were used for the IC₅₀ experiment. Analysis of the graph shows that the IC₅₀ value of linoleic acid is 5906 µM (Fig. 5a). To gain a better understanding of the inhibitory mechanisms associated with linoleic acid, the activity of BChE was examined with three different concentrations of linoleic acid (500–2500 µM). Linear regression analysis of the Michealis-Menten diagram, presented as a Lineweaver-Burk plot (Fig. 5b), revealed an *R*^2^ value of 0.963. The kinetic behavior of linoleic acid revealed a noncompetitive inhibitory activity characterized by a decrease in Vmax values and no effect on Km. Ki and Km values for linoleic acid were estimated to be 1400 µM and 145.7 µM, respectively. The docking results were consistent with our kinetic results, suggesting that linoleic acid binds to the allosteric site of BChE rather than competing with the substrates for the active sites on the enzyme surface (Fig. 5c, d). When the ∆G values for these sites were calculated, it was discovered that linoleic acid had a higher affinity for a hydrophobic pocket of the allosteric site with ∆G values − 7.8 and − 38.1 kcal/mol for the active and allosteric site of the BChE enzyme, respectively (Table 1).

**Fig. 6:**
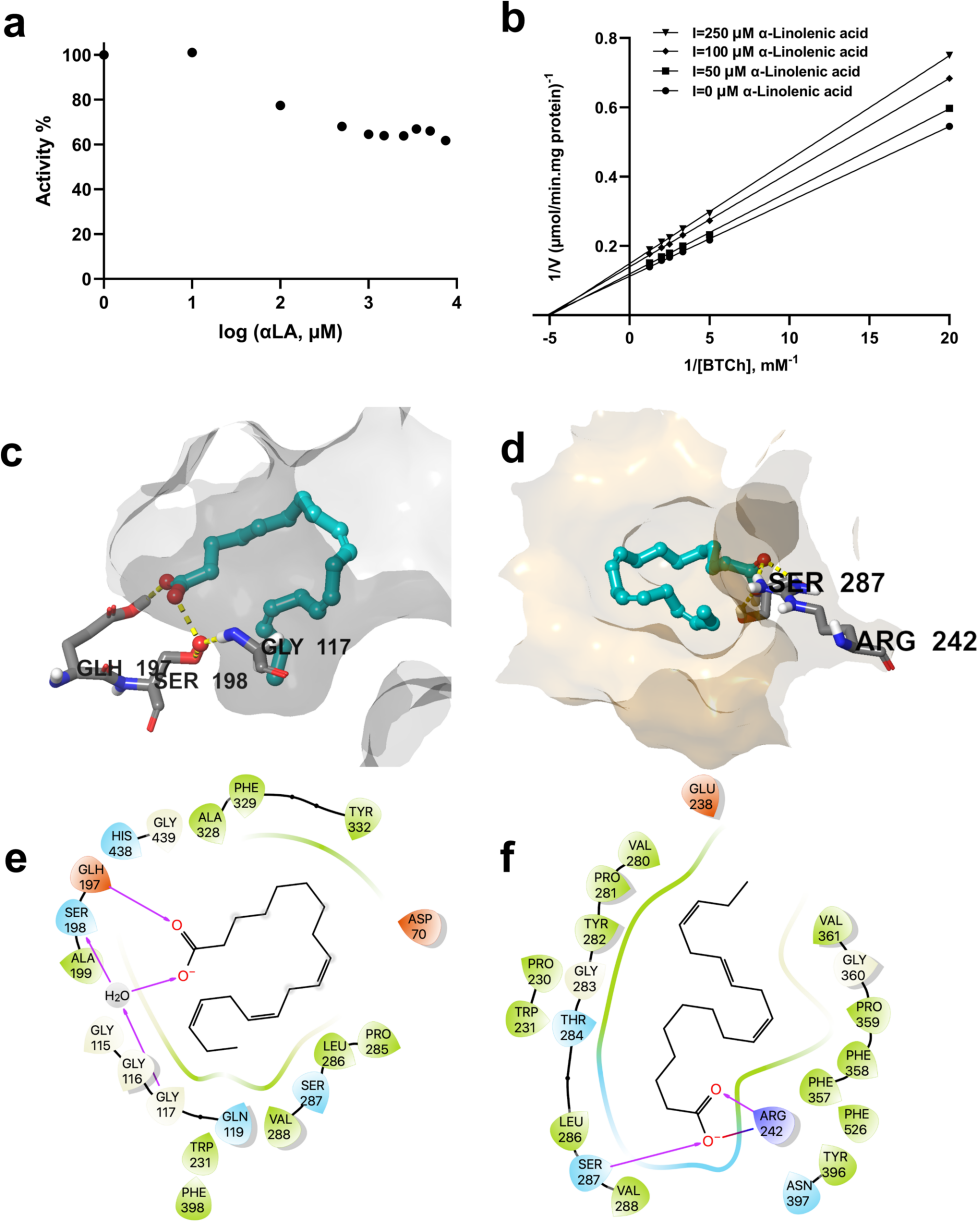
Kinetic characteristics and predicted binding interactions of α-linolenic acid with BChE. α-LA noncompetitively inhibits BChE and shows an IC₅₀ of 5810 µM and a Ki of 666.9 µM (**a**) and noncompetitive inhibition (**b**) of BChE by α-LA. Predicted binding of α-LA in the active site (**c**) and predicted allosteric site (**d**) of BChE (α-LA is shown in teal ball-and-stick representation, amino acid residues as gray sticks, water molecules as red spheres, and H bond interactions as yellow dashed lines). 2D interaction diagrams of α-LA for its predicted binding modes with BChE (**e** and **f**, respectively)

The IC₅₀ experiment for α-LA was performed with different doses (10–7500 µM). Using the activity % vs. concentration graph, the IC₅₀ value for α-LA was determined at 5810 µM (Fig. 6a). The inhibition mechanisms of α-LA were evaluated in the presence of three concentrations of α-LA (50–250 µM). Linear regression analysis of the graph presented as a Lineweaver-Burk plot with a goodness-of-fit value of *R*^2^ 0.983 (Fig. 6b). The addition of α-LA led to a decrease in Vmax values but had no effect on Km, indicating noncompetitive inhibitory behavior. The graph shows 666.9 µM and 184.8 µM for Ki and Km, respectively. Both the kinetic and docking results consistently showed that α-LA preferentially binds the allosteric site of BChE instead of the active site of the enzyme (Fig. 6c, d). α-LA showed a greater affinity for the allosteric site with a ∆G of − 49.5 kcal/mol compared to the active site (∆G =  − 10.9 kcal/mol) (Table 1).

Upon docking the fatty acids to the BChE active site and allosteric site, the fatty acids obtained better docking scores in the active site than the allosteric site for their selected poses (Table 1). However, better free binding energy values were obtained for the allosteric site when MM-GBSA calculations were performed. In the active site, the fatty acids were predicted to fit in the acylation site (A-site) making electrostatic contacts through their carboxylate head with the catalytic Ser198 residue, as well as A-site residues such as Gly117 and Glu197, and the water molecules intermediating between the catalytic and A-site residues (Figs. 2c, 4c, 5c, and 6c). The fatty acids were predicted to bind in a bent orientation to fit in the allosteric pocket except oleic acid, which stretched further to reach an adjacent pocket in the allosteric site (Fig. 2d, 4d, 5d, and 6d). The carboxylate head of the fatty acids, except arachidonic acid, was bound by electrostatic attractions to Arg242 located between the allosteric pocket and the entrance of the active site. The affinity of the compounds to the active site was lower than to the allosteric site of the enzyme, where the pseudo-substrate BCh should have a much higher affinity (Table 1). The table below presents the Km values for fatty acids inhibition types and kinetic parameters with BChE, using BTCh as substrate (Table 2).

**Table 2:** Inhibition types and kinetic parameters of fatty acids with BChE using BTCh as substrate, the Km values are given below

Compound	IC₅₀	Ki (µM)	Km (µM)	Inhibition type	Goodness of fit (*R*^2^)
Oleic acid (18:1)	2373	321.4	264.6	Noncompetitive	0.9571
Linoleic acid (18:2)	5906	1400	145.7	Noncompetitive	0.9630
α-Linolenic acid (18:3)	5810	666.9	184.8	Noncompetitive	0.9833
Arachidonic acid (20:4)	611	465.4	297.3	Uncompetitive	0.9817

## Discussion

This study explores the potential of different lipids to act as substrates or regulators of BChE and shows that both 18- and 20-carbon fatty acids with different levels of saturation have different effects on enzyme activity. Exploring the relationship between BChE and lipid metabolism could pave the way for therapeutic strategies for diseases associated with elevated BChE levels.

The role of BChE in lipid metabolism introduces another level of complexity (Gok et al. 2024). Lipid levels can be regulated by BChE, which breaks down choline esters produced during the breakdown and synthesis of fatty acids (Furtado-Alle et al. 2023). Studies on coconut oil intake show a link between increased BChE activity and increased triglyceride levels and lipid metabolism (de la Rubia Orti et al. 2021). This association underscores the role of BChE as both a regulator and responder in metabolic processes that potentially influence obesity, diabetes, and related diseases. Based on these studies, we investigated how fatty acids-specifically oleic acid, linoleic acid, alpha-linolenic acid, and arachidonic acid bind to BChE and influence its activity.

A more recent study has shown that AChE, a sister enzyme of BChE, is also inhibited by these fatty acids except for OA (Akay et al. 2023). In this study, trans-mono-unsaturated fatty acids were found to be more potent AChE inhibitors than saturated fatty acids. In addition, the 20-carbon fatty acid eicosanoic acid strongly inhibits AChE, indicating that chain length and unsaturation play a role. Among the fatty acids tested, AA exhibited the strongest inhibitory effect on AChE (IC₅₀), followed by LA and α-LA. Conversely, the noncompetitive inhibitory constants (Ki) show the opposite trend; Α-LA has the lowest Ki, indicating the strongest binding. In our results, we saw a similar noncompetitive inhibition type in LA and α-LA, with the exception of AA, which showed uncompetitive type inhibition. Similarly, AA displayed the strongest inhibitory activity (IC₅₀) against BChE compared to OA, α-LA, and LA, respectively. In contrast, the order of noncompetitive inhibitory constants (Ki) showed that OA had the lowest Ki value and thus the highest binding affinity. The results of molecular modeling were consistent with the in vitro results. An allosteric ligand binding site for BChE was suggested for the first time, to which the fatty acids were predicted to show affinity. The structural differences between AChE and BChE, which contribute to their unique substrate specificities and inhibition of the enzyme, require significantly increased concentrations of the compound, indicating a concentration-dependent modulatory function. In the molecular modeling experiments in the study by Akay et al., the binding of fatty acids to the human AChE active site was predicted to be quite different from that in the BChE active site in this study. The carboxylate head of the fatty acids is predicted to be located in the A-site close to the catalytic triad in the BChE active site, whereas it is predicted to bind to the residues in the P-site and near the entrance of the active site of AChE. In addition, the docking results indicate a better affinity of these fatty acids to AChE than to BChE, which is consistent with the IC₅₀ values (Akay et al. 2023).

In this study, the Ki and IC₅₀ values of four different BChE inhibitors were determined and compared. The type of inhibition, either noncompetitive or uncompetitive, depends on the specific interactions between the fatty acid and the enzyme. Noncompetitive inhibition of fatty acids occurs when a fatty acid binds to the allosteric site independently of the presence of a substrate. It is classified as uncompetitive when binding solely to the enzyme-substrate complex (Segel 1975). It becomes clear that these two measured values, which are used to evaluate the efficacy of inhibitors, have different meanings in context. Ki defines the equilibrium constant of the inhibitor-enzyme complex and reflects the binding affinity of the inhibitor to the enzyme. In contrast, IC₅₀ measures the concentration of an inhibitor required to reduce the reaction rate by half under certain experimental conditions. For both competitive inhibition and noncompetitive inhibitors, IC₅₀ values are generally higher than Ki values (Burlingham and Widlanski 2003). The data obtained show that the IC₅₀ values for all inhibitors are greater than the Ki values. This emphasizes the contextual differences between IC₅₀ and Ki measurements and shows that the experimental conditions have a significant influence on the IC₅₀. For linoleic and linolenic acids in particular, the large differences between IC₅₀ and Ki indicate that despite their high binding affinity, higher concentrations are required for effective inhibition. In the case of arachidonic acid, the relatively smaller difference between IC₅₀ and Ki suggests that the inhibitory effect and binding affinity are more balanced. These results emphasize the importance of considering experimental conditions in BChE enzyme inhibition studies and highlight the importance of understanding the relationship between Ki and IC₅₀ in the development and evaluation of inhibitors.

Inhibition of BChE by unsaturated fatty acids has several potential physiological consequences, possibly affecting lipid and energy regulation. Since BChE interacts with lipid metabolism, changes in BChE activity may affect obesity and metabolic syndrome, which depend on lipid regulation (Li et al. 2008). In addition, BChE plays a role in neurodegenerative diseases such as Alzheimer’s disease (AD) by influencing cholinergic neurotransmission through the hydrolysis of acetylcholine. Inhibition of BChE by unsaturated fatty acids might offer neuroprotection by extending acetylcholine’s action, potentially attenuating the cognitive decline associated with AD. Furthermore, since BChE is associated with systemic inflammation that occurs in many chronic diseases (Silman 2021), its modulation by unsaturated fatty acids could influence inflammatory responses.

The link between BChE, metabolic disorders and neurological diseases such as Alzheimer’s disease underscores its potential therapeutic role. The ability of lipid-lowering drugs such as statins to inhibit BChE suggests a potential role in dementia prevention (Darvesh et al. 2004). The dual action of statins on lipid metabolism and the cholinergic system offers a promising avenue for the treatment of neurodegenerative diseases. The unusual role of BChE in lipid metabolism suggests an important regulatory function, mainly affecting enzyme and lipoprotein concentrations. The metabolic context may also affect these primary functions, but further research is needed to confirm this and quantify the effect (Bulut et al. 2013; Furtado-Alle et al. 2023).

BChE hydrolyzes ghrelin, a hormone that has a significant influence on stress and hunger regulation (Schopfer et al. 2015). Another study showed that BChE knockout mice become obese when fed a high-fat diet (Li et al. 2008). Our previous research has shown that BChE can hydrolyze 4-mu-palmitate and that changes in LA and α-LA levels affect BChE levels in HepG2 cells (Gok et al. 2016, 2023). This research is also uncovering new dimensions of enzyme regulation and therapeutic possibilities in the relationship between cholinesterases, fatty acids, and their by-products. Future research, particularly using neuronal and hepatic cells, should investigate the effects of these fatty acids on BChE activity to clarify their physiological significance. To explore the overall systemic effects and potential therapeutic benefits of dietary fatty acids on cognitive function in Alzheimer’s disease, Alzheimer’s disease models could demonstrate whether dietary fatty acids could contribute to the maintenance of cognitive function. Research into unsaturated fatty acids could provide important insights into their therapeutic potential in metabolic and neurodegenerative diseases. The diverse influence of AChE and BChE, ranging from modulation of acetylcholine signaling to lipid metabolism and emotion regulation, offers exciting possibilities for advances in medicine and biochemistry.

## Conclusion

This study has shed light on the interactions of BChE with fatty acids and its unique role in metabolic processes, allowing us to better understand the mechanisms of enzyme inhibition and binding affinity. The regulatory effects of fatty acids on BChE and AChE emphasize their potential as therapeutic targets for neurological and metabolic diseases. In particular, the dual action of statins and the effects of BChE on lipid metabolism and the neurological system offer new strategies for the prevention or treatment of Alzheimer’s disease and other neurodegenerative disorders. However, more comprehensive studies are needed to better understand these relationships and translate them into clinical applications. The complex role of BChE, encompassing enzyme regulation, metabolic health and neurological function, offers a broad spectrum of potential impact and represents a promising avenue for future research.

## Data Availability

All source data for this work (or generated in this study) are available upon reasonable request.
